# Global Screening of Human Cord Blood Proteomes for Biomarkers of Toxic Exposure and Effect

**DOI:** 10.1289/ehp.11816

**Published:** 2008-12-02

**Authors:** David R. Colquhoun, Lynn R. Goldman, Robert N. Cole, Marjan Gucek, Malini Mansharamani, Frank R. Witter, Benjamin J. Apelberg, Rolf U. Halden

**Affiliations:** 1 Department of Environmental Health Sciences, Johns Hopkins Bloomberg School of Public Health, Baltimore, Maryland, USA;; 2 Mass Spectrometry and Proteomics Facility, Institute for Basic Biomedical Sciences and; 3 Department of Gynecology and Obstetrics, School of Medicine, Johns Hopkins University, Baltimore, Maryland, USA;; 4 Department of Epidemiology, Johns Hopkins Bloomberg School of Public Health, Baltimore, Maryland, USA;; 5 Center for Environmental Biotechnology, Biodesign Institute, Arizona State University, Tempe, Arizona, USA

**Keywords:** cigarette smoke, comparative proteomics, iTRAQ, umbilical cord, serum

## Abstract

**Background:**

Exposures of pregnant women to natural and manmade chemicals can lead to negative health effects in the baby, ranging from low birth weight to developmental defects. In some cases, diseases were postulated to have their basis in toxic exposure *in utero* or in early childhood. Therefore, an understanding of fetal responses to environmental exposures is essential. To that end, cord blood is a readily accessible biofluid whose proteomic makeup remains mostly unexplored when compared with that of adults.

**Objectives:**

Our goal was an initial global assessment of the fetal serum proteome and for the identification of protein biomarkers indicative of toxic *in utero* exposures related to maternal cigarette smoking.

**Methods:**

Drawing from a repository of 300 samples, we selected umbilical cord blood sera from 12 babies born to six smokers and six nonsmokers and analyzed both sample pools by tandem mass spectrometry in conjunction with isobaric tags (iTRAQ) for protein quantification.

**Results:**

We identified 203 proteins, 17 of which were differentially expressed between the cigarette smoke–exposed and control populations. Most of the identified candidate biomarkers were biologically plausible, thereby underscoring the feasibility of screening neonates with global proteomic techniques for biomarkers of exposure and early biological effects triggered by *in utero* chemical exposures.

**Conclusions:**

This validation study provides an initial view of the proteome of human cord blood sera; it demonstrates the feasibility of identifying therein by use of proteomics, biomarkers of environmental, toxic exposures.

Environmental and occupational exposures of pregnant women may lead to negative health effects ranging from low birth weight to developmental defects in the baby (Buczynska and Tarkowski 2005). In some cases, long-term effects, such as diabetes and chronic heart disease, are postulated to have their basis in fetal and early childhood exposure and development (Barker et al. 2002). Therefore, an understanding of fetal responses to environmental exposures is of increasing importance. If exposures occurred close to birth, and/or if the exposures are to substances with long half-lives that persist as a body burden through pregnancies, direct measurement of environmental agents in umbilical cord blood can be useful. However, because we are often most interested in effects of labile stressors at earlier stages in pregnancy (during organogenesis), the temporal aspect of toxic assault may best be dealt with by identifying biomarkers of effect that can indicate enduring biological changes that suggest both historical exposures throughout gestation and potential future adverse health outcomes.

Inhaled cigarette smoke is a common and high-quantity environmental exposure involving a complex mixture of multiple toxic substances, including carcinogens and mutagens. Directly inhaled mainstream cigarette smoke contains > 4,000 compounds (Rodgman et al. 2000). The relationship between smoking and poor health is well established (Armour et al. 2005; Keys et al. 2004), as are the health effects in neonates of mothers who smoke during pregnancy (Beratis et al. 1999; Bjørke Monsen et al. 2004; Department of Health and Human Services 2004). Because 10.7% of women in the United States smoke during pregnancy (Martin et al. 2005), the at-risk population of about 420,000 newborn infants each year is relatively high in the United States and, of course, much higher globally. Documented negative impacts on the fetus resulting from maternal cigarette smoking include preterm delivery and shortened gestation, fetal growth restriction and low birth weight, and sudden infant death syndrome (Department of Health and Human Services 2004). Other less well established adverse effects include later increased risk of type II diabetes, obesity, asthma, and impaired cognitive development (DiFranza et al. 2004; Gilliland et al. 2001; Montgomery and Ekbom 2002; Von Kries et al. 2002). Whereas cotinine in umbilical cord blood is a useful indicator for exposure to maternal active and passive smoking (Pichini et al. 2000), *in utero* biomarkers of toxic effects from cigarette smoke are still lacking.

Although genetic methods represent a relatively straightforward tool of testing for heritable diseases and susceptibility, protein biomarkers appear particularly well suited for measuring and detecting phenotypic manifestations of exposure and disease. For epidemiologic studies and clinical applications, proteomic mining strategies for biomarker discovery have focused on blood serum and plasma, because this compartment is relatively accessible and potentially provides a host of diagnostic information (Lathrop et al. 2003). Mass spectrometry (MS) is at the forefront of proteomic technologies for the global analysis of complex specimens such as human serum, which displays a high and thus challenging dynamic range with respect to protein abundance (Aebersold and Mann 2003; Anderson and Anderson 2002). This challenge is typically addressed by using prefractionation steps (e.g., depletion, precipitation, and strong cation exchange) before mass spectrometric analysis for enhancing the range of proteins detected (Li et al. 2005). Furthermore, isobaric tags for relative and absolute quantitation (iTRAQ) reagents (Ross et al. 2004) make it possible to quantitatively screen the entire proteome within the detectable dynamic range for qualitative and quantitative differences in protein expression between individuals and groups differing in exposure history, health status and disease.

Here we describe the application of quantitative tandem mass spectrometry (MS/MS) for the global, nontargeted characterization of the fetal cord blood serum proteome—an important task that has not yet been documented in the peer-reviewed literature. Our goal was to screen neonates for candidate biomarkers indicative of early biological responses to toxic exposures from smoke constituents *in utero*. Once obtained, an initial “snapshot” of proteins expressed in umbilical cord blood serum may serve as the starting point for developing low-cost assays for biomarkers of exposure and disease in this easily accessible and presumably information rich biofluid.

## Methods

### Sample selection and collection

Samples were collected from a subset of all singleton births that occurred from November 2004 to March 2005 at the Johns Hopkins Hospital, Baltimore, Maryland. This study required the collection of specimens that otherwise would have been discarded, and used information from medical records that was available to hospital personnel. The study received a HIPAA (Health Insurance Portability and Accountability Act) waiver. There was no requirement for informed consent because all samples and data were anonymous. All experimental protocols were approved by the Johns Hopkins Medicine Institutional Review Board (IRB) before sample collection under IRB approval number 04-04-22-02. A total of 341 samples were collected, of which 300 met information and volume criteria for the larger study. Human umbilical cord serum samples were collected immediately after birth using the Witter cord cradle (Witter et al. 2001), and stored at 4°C for < 3 hr before further processing. Serum samples were centrifuged at 1,000 × *g* for 15 min in a bench top centrifuge (Fisher Scientific, Palo Alto, CA). Aliquots were placed at −80°C for long-term storage. Measurement of cotinine was conducted as previously described (Bernert et al. 1997). Briefly, after sample extraction preparation, we analyzed serum using positive-ion atmospheric pressure chemical ionization MS/MS. We identified cotinine by multiple reaction monitoring of the characteristic mass transition, *m/z* 177 → 80, compared with a deuterated internal standard. In addition, we abstracted data from medical charts of mothers and infants relevant to smoking, birth outcomes, and health problems during pregnancy, labor, and delivery (Table 1).

From the abstracted data, we identified a subset of six infants of self-reported maternal smokers whose smoking status was verified by umbilical serum cotinine levels of > 10 ng/mL (representing active smokers) and six controls, identified as infants of self-reported nonsmokers with cotinine levels of less than the detection limit of 0.015 ng/mL. Self-reported nonsmokers with cotinine levels above the limit of detection were excluded from this study. All selected infants were term (≥ 37 weeks by obstetrical estimate) African-American males born by normal spontaneous vaginal delivery and having no known preexisting health conditions of parent and/or child, no known recreational and prescription drug use, and no acute infection of the mother, as noted on the records abstracted from the medical chart, and sufficient serum for analysis (complete cohort information is available online; THREE Study 2008).

### Protein purification and depletion

Stored serum (−80°C) was thawed to room temperature and aliquots of 100 μL were removed for processing. Samples were diluted to 500 μL using phosphate-buffered saline (PBS). The diluted samples were filtered through a 0.22-μm Spin-X filter (Sigma Chemical Co., St. Louis, MO) at 3,000 × *g* for 30 sec. A 100-μL sample was applied onto a ProteoPrep Top 20 protein immunoaffinity column (Sigma), which had previously been washed and equilibrated using PBS. Samples were incubated at room temperature for approximately 30 min, and then centrifuged at 2,000 × *g* for 30 sec to elute the depleted proteins. Immunoaffinity columns were washed with PBS twice, and these fractions were pooled with the initial flow-through, resulting in a pool of ~ 1.5–2 mL for each subject; each pool was concentrated to approximately 100 μL using Ultrafree MC 5 kDa molecular weight cutoff filters (Sigma). Bound proteins (the “top 20 proteins,” not used in this study) were eluted per the manufacturer’s directions to recharge the columns. Columns were then washed, re-equilibrated, and reused for multiple samples. We determined protein concentrations at each step using the bicinchoninic acid (BCA) assay (Pierce Biotechnology, Rockford, IL) to assess protein depletions. We assayed success of depletion (~ 98%) of major proteins by visualizing less abundant ones on silver-stained gels (data not shown).

### SDS-PAGE

During all steps, we assessed sample quality and reproducibility by sodium docecyl sulfate polyacrylamide gel electrophoresis (SDS-PAGE) using 4–12% Tris-HCl gels (Bio-Rad, Hercules, CA). Protein samples (approximately 2 μg) were run at 150 V for ~ 1 hr in 1X Tris-glycine-SDS buffer (BioRad) using the BioRad mini-Protean vertical electrophoresis system. Staining was completed using a published silver stain protocol (Shevchenko et al. 1996), and gels were imaged at 500 dpi using a Umax PowerLook 3 scanner with MagicScan software (version 4.5; Techville, Inc., Dallas, TX).

### Protein digestion and iTRAQ labeling

Before analysis, two pools were formed by combining the sera of babies born to smokers and those of babies born to nonsmoking mothers. Pooled samples were prepared in duplicate, thereby producing a total of 4 samples (2 + 2), each containing approximately 100 μg of protein (smoker pool and nonsmoker pool). These four pools were subjected to precipitation using 10% trichloroacetic acid in acetone (−20°C, overnight) followed by an acetone wash (−20°C, 15 min) and resuspension in 20 μL of 0.5 M triethyl ammonium bicarbonate. Duplicate composite samples of exposed and controls were subjected to iTRAQ labeling to produce technical replicates.

The pooled smokers and nonsmokers purified and depleted serum proteomes were reduced at 60°C for 1 hr with 2 μL 50 mM tris(2-carboxyethyl)phosphine (TCEP), and subsequently alkylated using 200 mM s-methyl methane thiosulfonate (MMTS) (1 μL) for 10 min. After digestion overnight at 37°C using 10 μL of 0.5 μg/μL porcine trypsin (Promega, Madison, WI), iTRAQ labeling was conducted for 1 hr at room temperature (Ross et al. 2004). The contents were then combined into a single tube and reduced to a volume of 50 μL.

### Strong cation exchange (SCX) chromatography

Chromatography by SCX was carried out using a PolyLC polysulfoethyl A column (10 cm, 2.1 mm, 5 μM particles, 300 Å pore) at a flow rate of 250 μL/min. We collected 22 fractions using a Probot fraction collector (Dionex, Sunnyvale, CA) along a 40-min gradient of 10 mM potassium phosphate in 20% acetonitrile and 350 mM potassium chloride in 10 mM potassium phosphate in 20% acetonitrile.

### LC-MS/MS analysis

After an initial survey of the cord serum proteome in non-depleted samples using nanospray liquid chromatography (LC) MS/MS, we performed all further analyses on SCX-fractionated proteomes using the same method. Briefly, peptides (5 μL) were separated on a C_18_ 75-μM column hand packed with YMC ODS-AQ (5 μM particle, 120 Å pore size) in 0.1% formic acid on a gradient (5–40%) using 0.1% formic acid/90% acetonitrile in 60 min, and a second, steeper gradient from 40–90% in 5 min. The flow rate was 300 nL/min. We conducted MS using an Applied Biosystems Q-Star Pulsar with a spray voltage of 2.2 kV (Applied Biosystems, Foster City, CA). We conducted a survey scan at *m/z* 350–1,200 and obtained subsequent data-dependent MS/MS scans for the three most intense ions using a 30-sec exclusion window. Data were analyzed using Protein Pilot, and mass spectra searched against the Swiss-Prot database release 52.5 (iTRAQ quantitative data) and Mascot database revision 070518 (nonquantitative; 4937571 sequences and 1702359384 residues) (Pappin et al. 1993) to determine log confidence scores for protein identification and to generate iTRAQ ratios (Applied Biosystems 2006). We validated the iTRAQ labeling by searching the data against the Swiss-Prot database using Mascot selecting iTRAQ modification as a variable modification. Quantitative data were exported from ProteinPilot to Excel (Microsoft, Bellevue, WA) and 95% confidence intervals (CIs) were generated for each ratio comparison (*m/z* 114:115, 114:116, 114:117; 115:114, 115:116, and 116:117). Each pairwise comparison was subjected to a two-tailed *t*-test to determine whether the ratio was different from 1 (*p* < 0.05). Only those proteins demonstrating significant (*p* < 0.05) quantitative differences in all four pairwise comparisons were considered to be regulated in response to maternal cigarette smoke exposure. Pairwise comparisons of the technical replicates resulted in the exclusion of statistically significant changes observed in only one technical replicate. The calculated *p*-values were exported into Q-value, and a false positive rate was estimated following the program’s instructions (Storey and Tibshirani 2003). Proteins matching cytokeratin were removed from the analysis because they were identified as laboratory contaminants based upon iTRAQ quantitative ratios.

### Western hybridization

Five milligrams each of samples were electrophoretically separated on a 4–12% NuPAGE gel. Thereafter, proteins were transferred to nitrocellulose filters at 50 V for 120 min in transfer buffer (50 mM Tris pH 7.5, 380 mM glycine, 0.1% SDS and 20% methanol). After blocking with TBS buffer (50 mM Tris, pH 7.5, 150 mM NaCl) containing 5% nonfat powdered milk (Safeway) and 0.1% Tween-20, filters were incubated at 4°C overnight with primary antibody. The following antibodies were used for immunoblotting: 1:1,000 dilution of anti-Gelsolin antibody (ab11081; AbCam, Cambridge, MA), which was selected as a control, because it showed a ratio equal to 1:1 in smoke-exposed and non-exposed individuals; 1:500 dilution of anti-α-fetoprotein (ab3980, AbCam), which was selected because it had a significant and high smoker:nonsmoker ratio. Blots were then washed three times (15 min each) in TBS-Tween, incubated with 1:5,000 dilution of horseradish peroxidase-conjugated goat anti-mouse or goat anti-rabbit antibodies (Pierce, Rockford, IL), and washed three times in TBS-Tween for 15 min each. Proteins were visualized by enhanced chemiluminescence (ECL) and exposure to Hyperfilm MP (Amersham Biosciences, Piscataway, NJ). We measured quantitative data using ImageQuant (GE Healthcare, Piscataway, NJ) using digital images of scanned films. The pixel intensity of each individual band was determined and corrected using the gelsolin intensity for the same sample; the sum of smoker band intensities was compared with the sum of nonsmoker band intensities. We used an unpaired, two-tailed *t*-test to test for differences between the two conditions.

## Results

**Figure 1:**
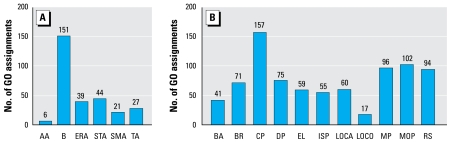
Gene ontology (GO) assignments for fetal proteins identified in cord blood sera. Proteins were grouped by molecular function (*A*) and biological processes (*B*). Use of the GOSt tool (Gene Group Functional Profiling 2008) resulted in the assignment of none, a single, or multiple molecular functions and biological processes for each protein examined. AA, antioxidant activity; B, binding; ERA, enzyme regulator activity; STA, signal transduction activity; SMA, structural molecule activity; TA, transporter activity; BA, biological adhesion; BR, biological regulation; CP, cellular processes; DP, development processes; EL, establishment of localization; ISP, immune system processes; LOCA, localization; LOCO, locomotion; MP, metabolic processes; MOP, multicellular organism processes; RS, responses to stimulus. Additional GO information and *p*-values can be found in the Supplemental Material, Table 3 (online at http://www.ehponline.org/members/2008/11816/suppl.pdf).

To characterize the umbilical cord proteome, we removed most of the most abundant 20 proteins and carried out two-dimensional LC-MS/MS coupled with iTRAQ labeling technology (Ross et al. 2004) on the depleted proteome. Database searches of acquired mass spectra yielded 203 unique proteins identified with ≥ 90% confidence; 201 of these were identified at the 95% confidence level [Supplemental Material, Table 1 (online at http://www.ehponline.org/members/2008/11816/suppl.pdf)]. Protein coverage ranged from 84% for vitamin D–binding protein to 9.6% for immunoglobulin (Ig) heavy chainV-I region Mot. Seventy-five percent of the proteins were classified as extra-cellular. These all had documented roles in binding or transport (Figure 1A). Because higher-order cellular processes are more varied, many proteins were assigned multiple potential biological roles in multiple processes (Figure 1B). No structural or other “leakage” proteins were identified.

**Figure 2:**
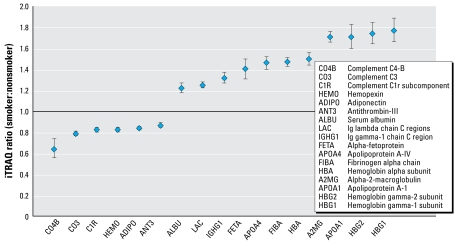
Quantitative information for fetal proteins identified as being significantly increased or decreased in response to maternal smoking (*p* < 0.05). Candidate biomarkers were identified at the ≥ 99% confidence level using at least two peptides. Quantitative values were converted to log values and the 95% confidence interval was calculated using pairwise comparisons between controls and smokers. Protein accession numbers, coverage information and statistical data can be found in Supplemental Material, Table 2 (online at http://www.ehponline.org/members/2008/11816/suppl.pdf).

To explore proteomic changes associated with environmental exposures, we conducted additional relative quantification analyses on sera from babies born to smokers and non-smokers. Representatives of both groups were very similar with regard to maternal gravida, age, education, marital status, and infants’ Apgar scores at 1 and 5 min. As might be expected, infants born to smokers were on average smaller and had shorter gestational age (see footnote in Table 1 for statistical information). Quantitative comparison of the protein expression in cord serum from infants of maternal smokers (average serum cotinine level of 187 ± 77 ng/mL) and nonsmokers (< 0.015 ng/mL) using a two-tailed *t*-test revealed 17 proteins whose expression was, in all four pairwise comparisons, statistically significantly up- or down-regulated in response to maternal smoking (*p* < 0.05). Statistical data estimated using Q-value suggested a false positive rate of < 0.01 or, in this case, less than one protein by chance alone [See Supplemental Material, Figure 1 (online at http://www.ehponline.org/members/2008/11816/suppl.pdf)]. Most of the identified candidate biomarkers shown in Figure 2 were abundant proteins. Relative abundance of proteins in serum from babies born to maternal smokers compared with maternal nonsmokers ranged from 77% increases (hemoglobin gamma-1 subunit) to 36% decreases (complement C4-B). Some changes were relatively small yet statistically significant, such as those observed for antithrombin-III (13% decrease) and complement C1r subcomponent (17% decrease). All proteins identified in our analyses were shared among babies born to smokers and nonsmokers; in other words, none of the detected proteins were unique to either study group. This is not surprising because these proteins have major structural and functional roles in human serum.

**Figure 3:**
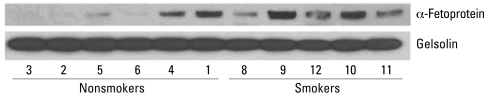
Western blot image confirming iTRAQ observed relative increases in α-fetoprotein levels in cord blood sera of babies born to maternal smokers versus control subjects. Gelsolin served as a reference protein unaffected by smoking behavior. Because sample volume was insufficient, data for the smoke-exposed individual 7 are missing.

We identified 17 candidate biomarkers of toxic *in utero* exposure (Table 2), 14 of which previously had been linked to cigarette smoking as determined in a literature review (Belch et al 1984; Calori et al. 1996; Capuano et al. 2006; Cervi and Feighery 1991; Craig et al. 1989; D’Souza et al. 1978; Fager et al. 1980; Goldenberg et al. 1991; Hsieh et al. 1984; Iscan et al. 1997; Kawada 2004; Kew et al. 1987; Kim et al. 2006; Koethe et al. 1995; Mercelina-Roumans et al. 1996, 1997; Steinmetz et al. 1988; Van Tiel et al. 2002; Yuan et al. 2007). Of these, 11 were previously described following targeted measurements in blood from newborns, whereas the remaining ones were from experimental models (e.g., purified proteins, animal studies) or from studies of adults who had either long-term, high-level exposures or diseases associated with cigarette smoke exposure. Additionally, Western hybridization of samples with monoclonal antibodies confirmed the qualitative and quantitative results of the iTRAQ measurements, and also produced evidence for significant inter-individual variability in fetal protein expression (Figure 3). Relative quantification of α-fetoprotein by Western hybridization showed a ratio of 1:1.54 (smokers to nonsmokers), which was statistically significant (*p* = 0.029) and comparable to the iTRAQ measurements (average ratio, 1:1.4; 95% CI, 1.31–1.5). Sample processing was performed with good consistency, as demonstrated by gelsolin measurements varying by only 6% between individual samples (1.012 ± 0.04).

We identified 17 candidate biomarkers of exposure and effect within the fetal blood serum proteome. Most of these were not only biologically plausible but also confirmed by prior reports on exposure-related changes in their expression levels (Table 2). Detection of this relatively small number of modulated proteins (17 of 203 total; ~ 8%) demonstrates that the chosen methodology was sufficiently discriminatory and stringent.

## Discussion

Maternal smoking has been associated with low birth weight and other developmental problems, but the underlying mechanisms of these responses are not yet fully understood. Cell metabolism interference (Hsieh et al. 1984), endocrine perturbation (Beratis et al. 1999), and hypoxia (Varvarigou et al. 1994) have all been postulated as potential mechanisms for the effects of smoking-related health defects. Our data suggest that babies exposed to cigarette smoke *in utero* exhibit some individual protein expression patterns similar to those previously observed in adult smoke-exposed subjects and animal models. Documented effects include changes in the complement system (Capuano et al. 2006; Kew et al. 1987; Koethe et al. 1995), α-2-macroglobulin (Belch et al 1984; Goldenberg et al. 1991), hemopexin and albumin (Ahlsten et al. 1989; Ingvarsson et al. 2007), all suggestive of inflammatory alterations in the neonate. Alterations in Ig “C” chains are consistent with earlier reports that infants of smoking mothers have higher levels of IgG (Cervi and Feighery 1991). Additionally detected were several indicators of altered lipid metabolism, specifically adiponectin (Kim et al. 2006; Yuan et al. 2007) and two apolipoproteins, A-I and A-IV (Craig et al. 1989; Fager et al. 1980; Steinmetz et al. 1988). Interestingly, although apolipoprotein A-I was reportedly decreased in cord blood of infants of smokers in a prior study, it was found to be up-regulated here.

There was one indicator of altered hemostasis (fibrinogen). Observed changes in α-fetoprotein levels may be interpreted as evidence for impaired fetal growth and maturation, both known to be associated with maternal smoking (Beratis et al. 1999). α-Fetoprotein is produced by the fetal liver. In the past, its presence in cord blood has been associated with decreased fetal growth, a known consequence of maternal smoking (Beratis et al. 1999). Lower levels of apoliprotein A-I found among infants of smoking mothers may indicate altered lipid metabolism (Iscan et al. 1997), whereas increased α-2-macroglobulin expression points to inflammatory processes. The observed differential levels of hemoglobin subunits may well be related to a previously described relationship between smoking and higher hemoglobin levels both in adults and in cord blood of infants of smoking mothers (D’Souza et al. 1978; Kawada 2004; Van Tiel et al. 2002).

The sample selection in this study was limited by a number of variables, first and foremost the availability of a sufficient quantity of serum for proteomic analysis. African Americans were selected as the study group because they represented nearly 70% of the population sample. Other selection criteria were specified to remove as many effect modifiers and confounding variables as possible (e.g., alcohol and drug use, delivery type). As a result of the aforementioned selection criteria, this study is not necessarily representative of a more diverse and heterogeneous population. Further studies of broader sample groups are required to determine whether the responses observed here can be generalized to other populations. However, the strict selection criteria in this study allowed for the identification of a number of proteins whose expression is changed in response to cigarette smoke exposure.

Global screening using the iTRAQ technique also led to the discovery and identification of candidate biomarkers not anticipated or predictable *a priori*. The latter include the measured reduction of adiponectin levels in the pool of babies born to smoking mothers. This is intriguing because adiponectin has been implicated in metabolic syndrome in adults (Tajtakova et al. 2006), which may well begin in early childhood (Hales and Barker 2001). The biological role and mechanism of this and other incompletely understood proteins warrants further investigation. The construction of protein pathways (i.e., analyzing the results of proteome-wide studies by examining the relationships between regulated proteins, rather than on an individual protein-to-protein basis) may help elucidate the mechanistic basis of observed exposure effects, not only to cigarette smoke constituents but also to other environmental pollutants. Furthermore, this analytical strategy may open novel avenues of investigation by generating physiologic hypotheses that later could be tested using immunoassays and other classical biochemical techniques.

Overall, our work provides new evidence for important protein expression changes occurring in the fetus in response to maternal smoking. Although protein expression changes would be expected (and some of these changes could be unimportant or caused by various confounding exposures and events), the very fact that many of the changes observed in this work are either biologically plausible or previously documented in mammalian systems (e.g., human babies, adults, and cell culture lines) suggests that the results are biologically relevant. The utility of this proteome-wide approach to exploring environmental exposures is that a broad screen in tandem with quantitative analysis can reveal a broader picture of the overall exposure effects than using traditional, targeted methods; however, validation and confirmation remain critical steps in this experimental pathway.

Because these analyses were conducted on pooled samples, interindividual variability may be a source of uncertainty. A fairly large increase or decrease would be required for any one individual level to affect the mean for the pool of six individuals. We saw no changes in iTRAQ ratios of > 1.8:1, however. Sample pooling is an accepted approach in proteomics; its benefits and limitations have been discussed elsewhere (Karp et al. 2005; Gan et al. 2007).

When working with low-volume samples, proteomics by necessity can achieve only limited coverage of the blood proteome, which contains thousands of proteins and spans a dynamic range of > 10 orders of magnitude (Anderson and Anderson 2002). Thus, the total spectrum of proteins contained in human blood certainly is much larger than what is reported here. Limitations are expected to arise from using low-volume samples and blood serum as opposed to plasma. Serum contains relatively fewer proteins but it is customarily collected and therefore more readily available. To capture low-abundance proteins such as interleukins, current methods require a large starting volume and extensive prefractionation for removal of more abundant proteins. Sample pooling can address this need when working with low-volume samples, such as umbilical cord blood serum. The coverage achieved in this study is comparable to prior work, in which between 70 and 300 proteins were identified by individual MS methods (e.g., Li et al. 2005; Stalder et al. 2008; Whiteaker et al. 2007). Given the low volume of serum used, the obtained coverage is noteworthy.

Further studies using Western hybridization and/or high-throughput MS methods may represent a logical and practical next investigative step. Levels of proteins also may be scrutinized on a person-by-person basis to help understand variability among individuals. Such research could evaluate effects of confounders that were selected out of our study but which may be important for capturing the full range of protein expression. Future proteomic studies could be combined with investigations of genetic traits (e.g., single nucleotide polymorphisms) and of epigenetic changes to develop fundamental knowledge about the role of cigarette smoke and other environmental stressors in adverse alterations of the phenotype.

An important yet unanswered question is whether the observed fetal responses to exposures from cigarette smoke constituents are enduring and indicative of deleterious health effects in early childhood and later in life. Prospective cohort studies could provide answers and help to further define the value of the umbilical cord blood serum proteome as a diagnostic window into human physiology and pathology. Furthermore, recently developed high-throughput protein biomarker screening methods, such as multiple reaction monitoring, could be applied to a broader cohort of samples for a further validation of the observed biological changes (Sandhu et al. 2008; Whiteaker et al. 2007). Because this method has been posited not to require abundant sample preparation, a prospective cohort could be rapidly assayed for the absolute presence of these biomarkers.

It has been theorized that a characterization of neonate exposures can lead to an understanding of general health outcomes (Barker et al. 2002). Using a well-characterized, high-magnitude exposure, differences in protein abundances related to that exposure were measured. This validation study yielded some interesting initial information on the composition of the human umbilical cord blood serum proteome and subsequent global measures of exposure effects upon that proteome. By leveraging MS technology, we demonstrated that the effects of environmental exposures at the protein level may be examined in previously unforeseen ways, giving the potential to address important biological issues from a systemwide viewpoint.

Therefore, the potential exists to expand the applications of high-throughput MS techniques for the comprehensive analysis of environmental exposures, ranging from the characterization of ecotoxicologic effects to the epidemiologic analysis of chemical exposures (Dowling and Sheehan 2006; Sheehan 2007). Given the positive experiences gained from this study, we posit that the potential for global screening for protein expression differences in cord blood repositories may aid in the investigation of infant health outcomes associated with *in utero* exposures to environmental toxicants.

## Figures and Tables

**Table 1:** Characteristics of nonsmoking and smoking mothers and their babies.

						Apgar score				
Subject	Gravida	Marital status	Age (years)	Education (years)	Alcohol	1 min	5 min	Birth weight (g)	Cotinine (ng/mL)	Gestational age (days)
Nonsmokers										

1	5	Single	29	11	No	9	9	4,044	0.0075^a^	287
2	2	Married	29	> 16	No	9	9	3,719	0.0075	280
3	3	Single	24	16	No	7	9	2,824	0.0075	286
4	2	Single	25	12	No	8	9	2,824	0.015	282
5	5	Single	38	12	No	9	9	3,199	0.0075	275
6	9	Single	28	10	No	9	9	3,851	0.0075	273
Mean	4	Single	29	13	No	9	9	3,410	0.0088	281

Smokers										

7	6	Single	27	12	No	9	9	2,919	125	279
8	4	Married	39	14	Missing	8	9	3,289	143	270
9	11	Married	36	12	No	9	9	3,566	368	269
10	7	Single	22	10	No	9	9	3,580	118	285
11	5	Married	33	15	No	4	6	2,050	229	272
12	2	Single	18	10	No	8	9	2,529	150	271
Mean	6	Single	29	12	No	8	9	2,989	189	274

No statistically significant differences were observed (*p* > 0.05). There was a trend for nonsmokers to have, on average, a 6-day longer gestational age (*p* = 0.053) and to be an average of 421 g heavier (*p* = 0.12).

a Assuming 1/2 limit of detection.

**Table 2:** Candidate biomarkers found to be modulated in response to maternal smoking.

Name	Literature findings	Study
Down-regulated in current study		

Adiponectin^a^	Decrease observed in mice transgenic for human APOb100 in active smoking model, and in male smokers	Kim et al. 2006; Yuan et al 2007
Antithrombin-III^a^	Nonsignificant lower levels in thrombin–antithrombin complex in newborns of smokers	Mercelina-Roumans et al. 1996, 1997
Complement C1r subcomponent^a^	50% consumption with exposure to TGP *in vivo*	Koethe et al. 1995
Complement C3^a^	“Significant decrease in serum C3 levels in smokers”	Capuano et al. 2006; Hsieh et al. 1984; Kew et al. 1987; Koethe et al. 1995
Complement C4-B^a^	20% consumption with exposure to tobacco glycoprotein in purified protein studies	Koethe et al. 1995
Hemopexin	ND	

Up-regulated in current study		

Albumin (serum)	NR	Ahlsten et al. 1989; Ingvarsson et al 2007
α-Fetoprotein^a^	Significantly increased in babies of maternal smokers compared with nonsmokers	Beratis et al. 1999
α-2-Macroglobulin^a^	Increased in serum of smokers and of newborns of smokers. Increased in maternal smokers and associated with fetal growth retardation in their infants	Belch et al 1984; Goldenberg et al. 1991
Apolipoprotein A-I^a^	Decrease by 4.2% in smokers vs. nonsmokers; decrease in infants of smoking mothers	Craig et al. 1989; Fager et al. 1980; Iscan et al. 1997
Apolipoprotein A-IV	NR	Steinmetz et al. 1988
Fibrinogen α chain^a^	Significant increases in smokers and heavy smokers, but no association in newborns	Belch et al. 1984; Calori et al. 1996; Hsieh et al. 1984; van der Salm et al. 1994
Hemoglobin α subunit	Increased hemoglobin levels in adult smokers and cord blood of smokers	D’Souza et al. 1978; Kawada 2004; Van Tiel et al. 2002
Hemoglobin γ-1	Increased hemoglobin levels in adult smokers and cord blood of smokers	D’Souza et al. 1978; Kawada 2004; Van Tiel et al. 2002
Hemoglobin γ-2 subunit	Increased hemoglobin levels in adult smokers and cord blood of smokers	D’Souza et al. 1978; Kawada 2004; Van Tiel et al. 2002
Ig γ-1 chain C	Increased IgG levels in cord blood in newborns	Cervi and Feighery 1991
Ig λ chain C	Increased IgG levels in cord blood in newborns	Cervi and Feighery 1991

Abbreviations: ND, do data found; NR, no evidence for consistent relationship to smoking.

a Proteins previously reported to vary in expression as a function of cigarette smoke.
